# Surgery

**Published:** 1860-01

**Authors:** S. W. Gross

**Affiliations:** Lecturer on Surgical Anatomy and Operative Surgery, Philadelphia


OR, BI-MONTHLY NOVELTIES IN MEDICAL, PATHOLOGICAL, SURGICAL, OBSTETRICAL, PHYSIOLOGICAL, AND CHEMICAL SCIENCE.

### SURGERY.

BY S. W. GROSS, M.D.,

LECTURER ON SURGICAL ANATOMY AND OPERATIVE SURGERY, PHILADELPHIA.

I.—1. *On Recto-Vesical Lithotomy, with the Report of a Case in which this Method was successfully employed.* By LOUIS BAUER, M.D., M.R.C.S., England, etc. (American Medical Gazette, September, 1859.)

2. *On a Case of Large Vesical Calculus, successfully removed by the Recto-Vesical Section.* By GEORGE SOUTHAM, Esq., Surgeon to the Manchester Royal Infirmary. (Transactions of the Royal Medical and Chirurgical Society. Lancet, September 3, 1859.)

DR. BAUER adopted, in his case, the recto-vesical operation of lithotomy for the removal of a calculus weighing an ounce and a half, and recommends it as being the most rational and preferable plan in all cases, excepting young children. Mr. Southam, on the other hand, instituted the procedure for the extraction of a calculus weighing nearly five ounces, but advocates the method only in those patients whose general health is much impaired, and in whom the bladder is indurated and contracted, and contains a stone of large dimensions.

With the latter operator, the great majority of surgeons agree that the lateral operation is the most feasible and attended with less difficulties than any other. Dr. Bauer is not wholly unsupported in his opinion; but very few surgeons of the present day will be found to think, with him, that recto-vesical lithotomy possesses advantages over all other methods.

The patient in the first case was twenty-six years of age, and in perfectly good health. A Sims's speculum was introduced into the rectum, which, together with the bladder, was opened behind the prostate gland. Some difficulty was experienced in extracting the calculus, and the wound was accurately approximated by five silver sutures. On the eighth day the stitches were removed, the wound was found to be thoroughly united, and the patient was dismissed.

The case of Mr. Southam occurred in a patient twenty-one years of age, who had been suffering with symptoms of vesical calculus for sixteen years. His general health was very much impaired, and the urine was loaded with pus and albumen. On an improvement in his condition, the operation was performed by dividing the sphincter and lower portion of the anus, and opening the urethra anterior to the prostate, the incision being afterwards prolonged through the gland and neck of the bladder, on account of the large size of the stone, which gave rise to some difficulty in its extraction. The patient made an excellent recovery, not, however, without the occurrence of a fistule in the membranous portion of the urethra, about a quarter of an inch long, which was closed by the application of nitrate of silver and the electric cautery.

Mr. Southam proposes to call his operation the recto-urethral.

II.—*Maisonneuve's Operation for the Removal of Naso-pharyngeal Fibrous Polyps* (Gazette Hebdom., Nos. 39, 40, pp. 609 and 625, September and October, 1859.)

The French medical world has lately been occupied in discussing an operation, proposed by M. Maisonneuve, for the extirpation of fibrous polyps involving the posterior nares and pharynx. The difficulties with which the surgeon has to contend, in reaching and completely removing the pedicle of these tumors, are well known to all operators; and, with a view to their obviation, the author advises the division of the soft palate in the median line, leaving intact its free border. Through this opening, which he denominates the *palatine button-hole*, he brings the tumor, and then, placing as far back on its pedicle as possible a loop of wire, he removes it by *écrasement*, afterwards closing the opening by points of suture or leaving it patent for further operations, if deemed necessary.

The principle on which this operation is founded is not a new one. In 1717, Manne, of Avignon, advocated the removal of fibrous tumors of the pharynx by dividing the soft palate in the median line, but he also included its free border in the incision. Nélaton also divided the soft palate, and with it a portion of the hard palate. Richard confined the incision to the hard palate, while Botrel proposed a modification of Nélaton's procedure by not including the free border of the soft palate. Dieffenbach seems to have been the first to propose leaving the free border intact; but he doubted its success, and reports only one favorable issue from this operation.

M. Maisonneuve has, therefore, merely revived an old operation, which had fallen into disuse, but which may prove of signal service, since the difficulty in this class of cases is not only in reaching the tumor, but in preventing its return, which may be effected by the judicious use of caustic applications. We thus infer that if the procedure be practiced, the wound should not be closed immediately, but that surgical interference be maintained until we are certain that the pedicle of the tumor be destroyed, a result absolutely necessary to a radical cure. In case of hemorrhage, the incision may be performed on one day and the tumor be removed the next; and it is stated that such wounds unite very readily.

III.—*Ligature of the Common Iliac Artery for Aneurism; use of Silver Ligature; Death on the twenty-sixth day, from exhaustion by Dysentery.* By WARREN STONE, M.D., Professor of Surgery in the University of Louisiana. (New Orleans Medical and Surgical Journal, p. 732, September, 1859.)

The subject of this operation was an Irishman, thirty-six years of age, who was admitted into the Charity Hospital, on account of a well-marked aneurism of the left external iliac and upper portion of the femoral arteries, its extent being two inches below and two inches and a half above the crural arch. The patient's general health was in a bad condition, the stomach being disordered, and the bowels much deranged, the discharges being of a dysenteric nature. Rest and appropriate treatment were resorted to, to improve his health, which were continued for six weeks, with the desired effect, and as the tumor suddenly enlarged, and inflammatory action was set up, the operation of ligating the left common iliac was resorted to.

The incision extended in the direction of the semilunar line from the upper border of the tumor to the cartilages of the ribs, and the tissues were separated in the usual manner. Some difficulty was experienced in detaching the peritoneum from the tumor to which it had become adherent, and in executing this step of the operation, this membrane was wounded to a slight extent. A silver wire was passed around the vessel and tied with only sufficient firmness to stop the current of blood. Its ends were cut off close to the knot, and the large wound was approximated by several points of the twisted suture.

The patient did well for several days; the circulation was readily re-established, and there was no tendency to gangrene. The dysentery, however, became worse, and the patient died on the twenty-sixth day after the operation. Unfortunately, an autopsy was not procured.

Contrary to the opinion of the majority of pathologists and surgeons, Dr. Stone states that it is not necessary to divide the internal and middle coats of an artery, and that when a vessel is ligated, adhesive inflammation never takes place on its inner surface. In accordance with his belief, he applied the silver ligature, which he considers harmless by its presence, with a view of merely approximating the sides of the vessel and stopping the current of blood without strangulating and producing ulceration of its coats. His conclusions are not borne out by his statements, nor does he tell us what will become of the silver wire.

IV.—*Amputation at the Hip-joint.* By A. H. BUCHANAN, M.D., Professor of Surgical and Pathological Anatomy and Physiology in the Medical Department of the University of Nashville. (Pamphlet, pp. 8, 1859.)

Professor Buchanan reports a successful case of amputation at the hip, the first operation of the kind ever performed in Tennessee. The patient, a lad fourteen years of age, was seized in June, 1859, with pain in the thigh, and when seen by the author, in a few weeks from this date, the limb was found to be the seat of extensive suppuration, for which an opening was made about four inches above the external condyle, and nearly a quart of very fetid pus was discharged. The swelling being thus diminished, a large osseous tumor was discovered at the middle and upper third of the thigh-bone, caused by inflammation and suppuration of the medullary canal and expansion of the outer compact layer of the bone. In September, spontaneous fracture of the bone had taken place near its middle, and the patient was much emaciated. Hectic fever had supervened, the limb was about four times the size of the sound one, and the seat of intense pain attended with the daily discharge of about eight ounces of thin, offensive, ichorous pus. On the fourteenth of September, on account of the great drain upon the system, the operation was performed.

A mixture of chloroform and ether having been administered, the limb was amputated by the antero-posterior flap method. When the anterior flap was made, there was a discharge of a large quantity of very offensive pus mixed with venous blood. Only six or eight ounces of blood were lost, and eleven arteries were ligated. Reaction took place in about four hours, and as the wound was well glazed, the flaps were brought together by means of the interrupted suture and adhesive strips, a tent having been interposed between the flaps, to allow of free drainage. Under the use of nutritious food, brandy, and porter, the lad made a good recovery, the wound having nearly cicatrized on the twenty-ninth day. The entire shaft of the thigh-bone was necrosed and broken about its centre.

V.—*A Case of Inguino-femoral Aneurism cured by Pressure on the External Iliac.* By W. R. GORE, M.D., Surgeon to the City of Limerick Infirmary. (Dublin Medical Press, vol. xiii., No. 1078, p. 131.)

The interesting case of Dr. Gore adds more confirmatory proof as to the beneficial results of alternating digital and instrumental compression—a method which has succeeded in effecting a cure in every case to which it has been applied. The period required for obtaining the happy issue was thirty-five days and twenty-two and a·half hours, which, although longer than is usually required, is not more tedious than the operation of ligating the extermal iliac, in which the external wound rarely perfectly cicatrizes in so short a time. This mode of treatment cannot be too much impressed upon the profession, and we trust to see it adopted in every case of aneurism.

The patient, an Irishman, twenty-four years old, was the subject of a large aneurismal tumor of the femoral artery, about an inch below Poupart's ligament, for which it was proposed to tie the external iliac, but symptoms of diabetes supervened, and it was abandoned for the safer method of compression. On the twenty-eighth of June, a bladder of ice was placed on the tumor, and digital compression was instituted upon the vessel as it passes under the crural arch. At the expiration of seventy-six hours the current of blood through the tumor was much lessened in strength, and the aneurism had become harder and diminished in size. The assistants becoming much fatigued, pressure by means of the finger was discontinued, and Carte's compressor was substituted. This alternated with digital compression for eight days, and was occasionally entirely given up on account of pain, the finger, however, producing less inconvenience than the instrument. The parts having become blistered and intolerant of pressure, the compressor was placed over the external iliac, but it was relieved by the finger, and occasionally by total rest. From the nineteenth of July the pressure was only made during the day, and was intermittent; on the fourteenth of August all pulsation had ceased.

A cure was thus effected in thirty-five days twenty-two hours and a half, by interrupted, intermittent digital and instrumental compression; and for one-half of this period the artery was not acted upon at all, and scarcely any for nine days before pulsation had ceased. The iliac and femoral above the sac, and the latter artery below this point, pulsated after the patient was dismissed.

VI. *Remarks upon a rare variety of Tumor of the Scrotal Coverings.* By M. A. VERUEUIL. (Gaz. Hebdom., No. 37, p. 581, September 16th, 1859.)

The author reports two cases of subcutaneous erectile venous tumors of the scrotum, increasing under the influence of warmth and much exercise, and diminishing by cold and rest. When extirpated, the growth is found to be composed of erectile venous tissue, of a dense structure, and even hard at certain points from plastic deposits, containing phlebolites, and a large number of cysts filled with serous or sero-sanguinolent fluid.

The conclusions of M. Verueuil are contained in the following summary:—

1. The scrotal veins, as those of other parts of the body, are liable to take on varicose enlargement.

2. These vessels are of three classes: one set supplying the testis, another ramifying in the subcutaneous cellular tissue, and the third belonging to the integuments.

3. The varicose condition may assume three distinct forms, referable to the three classes of veins.

4. Two of these forms are well known, constituting varicocele and cirsocele, which are observed separately or combined. They represent deep and superficial varix of the lower extremity, of which the former is by far the most frequent. The same may be said of varix of the scrotum.

5. The third form, which may be combined with either of the two preceding, is not sufficiently known; it is seated in the subcutaneous veins of the scrotum, and resembles erectile venous tumors situated in deeper parts.

6. They may be congenital, or appear during infancy, and may remain stationary for a number of years. Being indolent, of moderate size, and without any tendency to ulceration, they do not excite attention, and only require palliative treatment.

7. They increase the already known number of morbid conditions of the scrotum, and on this account merit special attention. In other respects they enter into the same general history of erectile tumors, which are often found at the period of birth.

8. On the approach of puberty these tumors show a great tendency to spontaneous inflammation. Their superficial position, doubtless, predisposes them to it, but the inflammation is benign, and readily yields to treatment. It, however, produces changes in the erectile tissue, rendering extirpation necessary, if an operation is decided upon.

9. This inflammation may be favorable if it attacks the whole tumor, by producing coagulation of the blood in all the vascular branches. On the other hand, it is equally as liable to produce injurious effects.

10. At the commencement, erectile, subcutaneous tumors of the scrotum may give rise to some difficulty in their diagnosis. The symptoms by which they can be recognized are—that they are seated in the scrotal coverings; that the cord and testis are not involved; the absence of pain; their slow progress; their soft consistence at the start; and the changes produced upon them by rest, cold, or warm applications. The diagnosis will be more easy if the skin presents varicosities, as it will after a time.

11. The prognosis is more serious if, on account of the increasing size of the tumor, an operation becomes necessary, since some danger always attends surgical interference in varicose enlargements. It will be better to operate before the tumor has attained a large volume, and during manhood, a period very favorable to the success of all operations.

12. If the tumor be small it may be destroyed by cauterization, or by means of Breschet's forceps, with angular branches. Coagulating injections, or the seton, are not proper. When of large dimensions, extirpation will be the only resource.

VII.—*On the Treatment of Epithelial Cancers by the Application of the Actual Cautery.* By M. Sédillot. (Revue de Thérapeutique Médico-Chirurgicale and Journal de Médecine de Bordeaux, October, 1859, p. 693.)

In the treatment of epithelioma, the rule, which is always observed, is to remove the whole of the morbid mass, together with a portion of the surrounding healthy tissues, in order to prevent a return, and this object may be effected by the use of the knife, the potential cautery, or by means of the Vienna, arsenical, or Canquoin's paste.

There are some cases which are very embarrassing to the surgeon, as it is difficult to stop the progress of the disease, and two dangers are thus encountered, either to abandon the patient to his fate or to expose him to great deformity, which is scarcely counterbalanced by the certainty of a cure. Cases of this description are those in which the cancroid affection threatens to involve the free border of the lids or attack the whole thickness of the walls of the nose, or when it approaches the commissure of the lips or the auditory canal.

It has long been known that fibrous tissue is not prone to be invaded by epithelial cancer, and M. Sédillot considers it philosophical to profit by this fact, and, by the application of the hot iron, produce a dense, fibrous tissue, with slight organization and no tendency to morbid changes. By this means to destroy the disease, and create a barrier to its extension with little danger of relapse.

Another motive for adopting this practice is, that prolonged suppuration is favorable to the elimination of cancerous elements, an opinion which the author has verified by the microscope, finding that portions of tissues, infiltrated with cancer at the time of the operation, did not present any trace of the disease after several weeks of suppuration. This was a favorite treatment of M. Boyer, who persisted in causing the wounds made in the extirpation of cancroid growths to suppurate for some time, with the effect of having but few returns.

M. Sédillot reports five cures by this procedure, of which the following case is a good example. A woman, seventy years of age, presented herself at the clinic with an epithelioma of the lower lip, of seven months duration, one and a third inches in breadth by one inch in length. The mucous membrane was scarcely ulcerated, but if the ordinary operation by a V-shaped incision had been performed, it would have been necessary to remove two-thirds of the lip. The most projecting portion of the tumor having been snipped off with the scissors, the hot iron was applied to it, and in four days more was again repeated. The cure was complete in fifteen days, and in two months after the operation a photograph of the patient was shown to the Academy of Sciences, from which it was evident that the middle portion of the lip was restored in a regular manner, its breadth and length being preserved, and the cicatrice being soft and unbroken.

The author believes the procedure to be very simple, not attended with much loss of substance, and more sure than the knife. As chloroform is always to be administered, the patient's fears are quieted, and he experiences no pain. Should an indurated spot appear in the cicatrice, denoting a tendency to a return of the disease, it must be treated in the same manner.

VIII.—*Ovariotomy in Ohio; being the Report of a Special Committee of the Ohio State Medical Society.* By J. W. HAMILTON, M.D., Special Committee on Surgery, Professor of Surgery in the Starling Medical College. (Ohio Medical and Surgical Journal, November, 1859, p. 91.)

This report embraces within its range fifty-one cases of ovariotomy performed in Ohio by surgeons of that and other States, and also cases operated upon by Ohio surgeons out of the State. It includes twenty-five hitherto unreported cases. From it we condense the following facts:—

1. The *age* of the patient is given in twenty-five instances, the average being thirty-four years, the extremes sixteen and fifty-six. The operation has been more successful in the younger than in the older, in the proportion of eight to five; that is, of twelve patients whose age averaged twenty-six and a half years eight were cured, while in the same number of the average age of forty-five and two-third years only five were cured.

2. *Tapping* does not appear to have compromised the chance of success, having been performed in nine cases from one to ten times. Subsequent extirpation resulted favorably in six of these.

3. *Adhesions* are specified in thirty-four cases; being slight or not existing at all in twelve, of which nine were cured. In twenty-three they were strong or extensive, and of these more than four-fifths were fatal. Absence of adhesions is, therefore, a favorable symptom.

4. The *length of the incision* is given in twenty-nine instances, in two of which the minor operation was performed. It ranged from six to twenty inches in thirty-seven cases.

5. *Separation of the ligatures* took place in eighteen cases in from fourteen to twenty-nine days, the average period being twenty-four days.

6. The *time of death* is specified in twenty-three instances. In two cases it occurred in one year; in one case in four and a half months; in three cases in from twenty to forty-five days; in five cases in from seven to seventeen days; in two cases in from five to six days; in six cases on the third day; in three cases within two days, and one patient died on the table.

7. The *cause of death* in fourteen cases is attributed in one to acute pneumonia; in two to gangrene of the sac; in one to congestion of the brain; in one to inanition; in one to shock; in one to exhaustion; in three to hemorrhage; and in four to peritonitis.

8. *Extirpation was impracticable* in thirteen instances. Of these seven died from the attempt within forty-five days; two lived one year; one was cured permanently; and in three the patient was rendered no better nor no worse.

9. *Extirpation was practicable* in thirty-seven cases, sixteen dying from the operation.

10. *Cures.*—In twenty-one cases recovery took place after the removal of the tumor, and, so far as has been ascertained, the patients are in good health, and there has been no return of the disease. In three the belly was opened, but on account of extensive adhesions the operation was abandoned. These cases recovered from the incision. *Thus we have twenty-four surviving, and twenty-seven perishing from the operation.* Of the former at least sixteen are now living and in good health.

11. The *size of the tumor* is specified in twenty-nine instances. The smallest two weighed five and seven pounds, the largest two one hundred and six and one hundred and thirty-six pounds. The average weight of all the tumors was thirty-four and two-third pounds. Further examination of these cases shows that extirpation of the larger tumors has been more favorable than that of the smaller, in the proportion of nearly three to one.

12. The *pathological characters* of forty-three tumors are specified. Thirty-two are cystic, the majority being compound; six are solid or fibrous; two omental; two uterine; and one a tubal fœtation.

Professor Hamilton concludes his interesting paper by discussing the propriety of receiving ovariotomy as an accredited operation, and concludes by saying that "the surgeon, ordinarily, has no right, till a less hazardous or more successful treatment is presented, to withhold the operation, when the diagnosis of cystic disease is clear, if, after a candid and full statement of its hazards, the patient is desirous to assume them." This remark is especially applicable to the proliferous cyst, on account of its rapid growth and its not admitting of cure by other means. Moreover, the author has shown that the compound cyst constitutes more than two-fifths of the forty-three specified tumors; the simple cyst amounting to only two-sevenths. Of the former eleven-sixteenths of the extirpations resulted in a cure; of the latter only one-third. Removal of the compound cyst is, therefore, a more successful procedure.

IX.—*New Operation for the Relief of the Pain in Acute Glaucoma.* By MR. HANCOCK. (London Lancet, October 29th, 1859, p. 435.)

Mr. Hancock, of the Royal Westminster Ophthalmic Hospital, has recently devised a new procedure for the relief of the pain of acute glaucoma, which consists in the introduction of a Beer's cataract knife at the junction of the sclerotic and cornea, and at the commencement of the lower semi-circumference of the latter. The blade of the instrument is inclined downward, and the point is inserted from the outer side of the eye, at the spot specified, passing inward and backward, traversing the coats of the cornea, and finally pushed toward the interior of the globe, dividing a portion of the ciliary ligament. It differs from Von Gräfe's operation, in that in his procedure a linear incision is made in the sclerotic, two-fifths of a line behind the cornea, and a sixth of the iris is removed.

The advantages of Mr. Hancock's incision are claimed to be :—

1. By the situation and oblique direction of the incision, a free drainage of the fluid is provided for.

2. The iris is very slightly wounded.

3. The pupil is preserved of its original size and in its normal situation.

4. The operation is very simple, and is performed remarkably quickly.

This method was practiced in a patient with acute glaucoma, in whom the globe of the eye was much enlarged, hard, and resisting, and who suffered severe pain when pressure was made on the parts. The operation had the effect of relieving these symptoms.

